# Apical approach in periodontal reconstructive surgery with enamel matrix derivate and enamel matrix derivate plus bone substitutes: a randomized, controlled clinical trial

**DOI:** 10.1007/s00784-021-04256-1

**Published:** 2021-11-17

**Authors:** Jose Antonio Moreno Rodríguez, Antonio José Ortiz Ruiz

**Affiliations:** 1 Murcia, Spain; 2 Department of Stomatology, Faculty of Medicine, University of Murcia, Murcia, Spain

**Keywords:** Clinical trial, Microsurgery, Periodontal regeneration, Periodontitis

## Abstract

**Objectives:**

This parallel, randomized controlled clinical trial evaluated the influence of bone substitutes (BS) on the efficacy of the non-incised papillae surgical approach (NIPSA) with enamel matrix derivate (EMD) in resolving deep, isolated, combined non-contained intrabony and supra-alveolar periodontal defects, preserving the soft tissue.

**Material and methods:**

Twenty-four patients were randomized to treatment with NIPSA and EMD or NIPSA plus EMD and BS. Bleeding on probing (BoP), interproximal clinical attachment level (CAL), interproximal probing depth (PD), recession (REC), location of the tip of the papilla (TP), and width of the keratinized tissue (KT) were evaluated before surgery and at 1 year post-surgery (primary outcomes). Wound closure was assessed at 1 week post‐surgery, and supra‐alveolar attachment gain (SUPRA-AG) was recorded at 1 year post‐surgery.

**Results:**

At 1 week, 87.5% of cases registered complete wound closure and there were no cases of necrosis, without differences between groups (*p* > .05)**.** At 1 year, all cases showed negative BoP. A significant PD reduction (NIPSA + EMD 8.25 ± 2.70 mm vs. NIPSA + EMD + BS 6.83 ± 0.81 mm) and CAL gain (NIPSA + EMD 8.33 ± 2.74 mm vs. NIPSA + EMD + BS 7.08 ± 2.68 mm) were observed (*p* < .001) in both groups, without significant between-group differences (*p* > .05). The residual PD was < 5 mm in all defects (NIPSA + EMD 2.50 ± 0.67 mm vs. NIPSA + EMD + BS 2.67 ± 0.78 mm). Soft tissues were preserved without significant between-group differences (REC: NIPSA + EMD 0.25 ± 0.45 mm vs. NIPSA + EMD + BS 0.17 ± 0.58 mm, *p* > .05; KT: 0.00 ± 0.43 mm vs. 0.08 ± 0.67 mm, *p* > .05). There were improvements in the papilla in both groups (TP: NIPSA + EMD 0.33 ± 0.49 mm vs. NIPSA + EMD + BS 0.45 ± 0.52 mm, *p* > .05), which was only significant in the NIPSA EMD + BS group (0.45 ± 0.52 mm; *p* < .05). In both groups, CAL gain was recorded in the supra-alveolar component, showing full resolution of the intrabony component of the defect in all cases (SUPRA-AG: NIPSA + EMD 1.83 ± 1.11 mm vs. NIPSA + EMD + BS 2.00 ± 1.76 mm, *p* > .05).

**Conclusions:**

NIPSA and EMD with or without BS seem to be a valid surgical approach in the treatment of isolated, deep non-contained periodontal defects. In our study, both treatments resulted in significant PD reduction and CAL gain, that extended in the supra-alveolar component, without differences with the use of BS. Both treatments resulted in soft tissue preservation. However, the addition of BS may improve interdental papillary tissue.

**Clinical relevance:**

NIPSA, with or without bone substitutes, resulted in significant periodontal improvement, with soft tissue preservation in isolated, deep non-contained periodontal defects. The application of bone substitutes may provide interproximal soft tissue gain.

**Clinical trial registration:**

Clinicaltrials.gov: NCT04712630.

**Supplementary Information:**

The online version contains supplementary material available at 10.1007/s00784-021-04256-1.

## Introduction

Periodontal disease is a bacterial biofilm caused chronic inflammatory disease that results in destruction of periodontal tissues and their disinsertion from the root surface [1–4]. As the destructiveness of periodontal tissues evolves coronoapically, disinserted periodontal tissues heal through epithelial apical migration of the gingival sulcus epithelium, forming the periodontal pocket [1, 5]. A periodontal lesions are configured by a periodontal pocket in the underlying bone, forming supra-alveolar or intrabony defects. In severe periodontal lesions, there is a deep interproximal clinical attachment loss that modulates the disease evolution, prognosis, and choice of treatment [6].

After non-surgical or surgical periodontal disease treatment, residual interproximal periodontal pockets or interproximal soft tissue defects condition the maintenance of the noninflamed periodontal status [7]. Residual defects may be unfavorable anatomically and a reservoir for dental plaque accumulation [8]. The resolution of interproximal periodontal pockets associated with papilla preservation and the reconstruction of supra-alveolar type defects are the major challenge and objective in periodontal reconstructive surgical treatment [6].

The non-incised papillae surgical approach (NIPSA) [9–11] is a micro-surgical technique for the treatment of isolated, deep periodontal defects and was developed to achieve optimal conditions for periodontal regeneration and soft tissue preservation. It involves a single incision in the mucosa, apical to the bone margin that delimits the periodontal defect, maintaining the structure of the marginal and interproximal soft tissue intact so that it acts as a “dome” protecting the space in the vertical component of the defect, and avoiding the collapse of the interproximal soft tissue in the underlying defect.

Studies of NIPSA have shown positive results in terms of periodontal pocket resolution, CAL gain, and soft tissue preservation [10, 11] and improvements in the interproximal soft tissue [11]. Because the use of bone substitutes (BS) is associated with NIPSA in all studies, it is difficult to determine how much BS helped to support the soft tissues, preventing their collapse over the defect, and improving papilla preservation.

The aim of this study was to evaluate NIPSA and EMD with (test procedure) and without BS (control procedure) in the resolution of isolated, deep combined non-contained intrabony and supra-alveolar periodontal defects with preservation of the papilla.

## Material and methods

### Trial design

We designed a double blind, parallel group, randomized, controlled, superiority clinical trial to assess the treatment of deep, isolated, non-contained periodontal defects after 1 year of evolution, using NIPSA combined with BS or not. The alternative hypothesis was that NIPSA and EMD with BS achieve better clinical results than NIPSA and EMD without BS. The study was carried out in a private clinic in Murcia, Spain, and commenced in September 2019. The study complied with the CONSORT statement on improving the quality of parallel-group randomized clinical trials. All clinical procedures were performed according to the Declaration of Helsinki and Good Clinical Practice Guidelines, as revised in 2013. The study protocol was approved by the Research Ethics Committee of the University of Murcia (Spain) (protocol number: 2441/2019) and was registered as a clinical trial (Clinicaltrials.gov) database (NCT04712630).

### Experimental groups: randomization, allocation and allocation concealment, and blinding

**Fig. 1:**
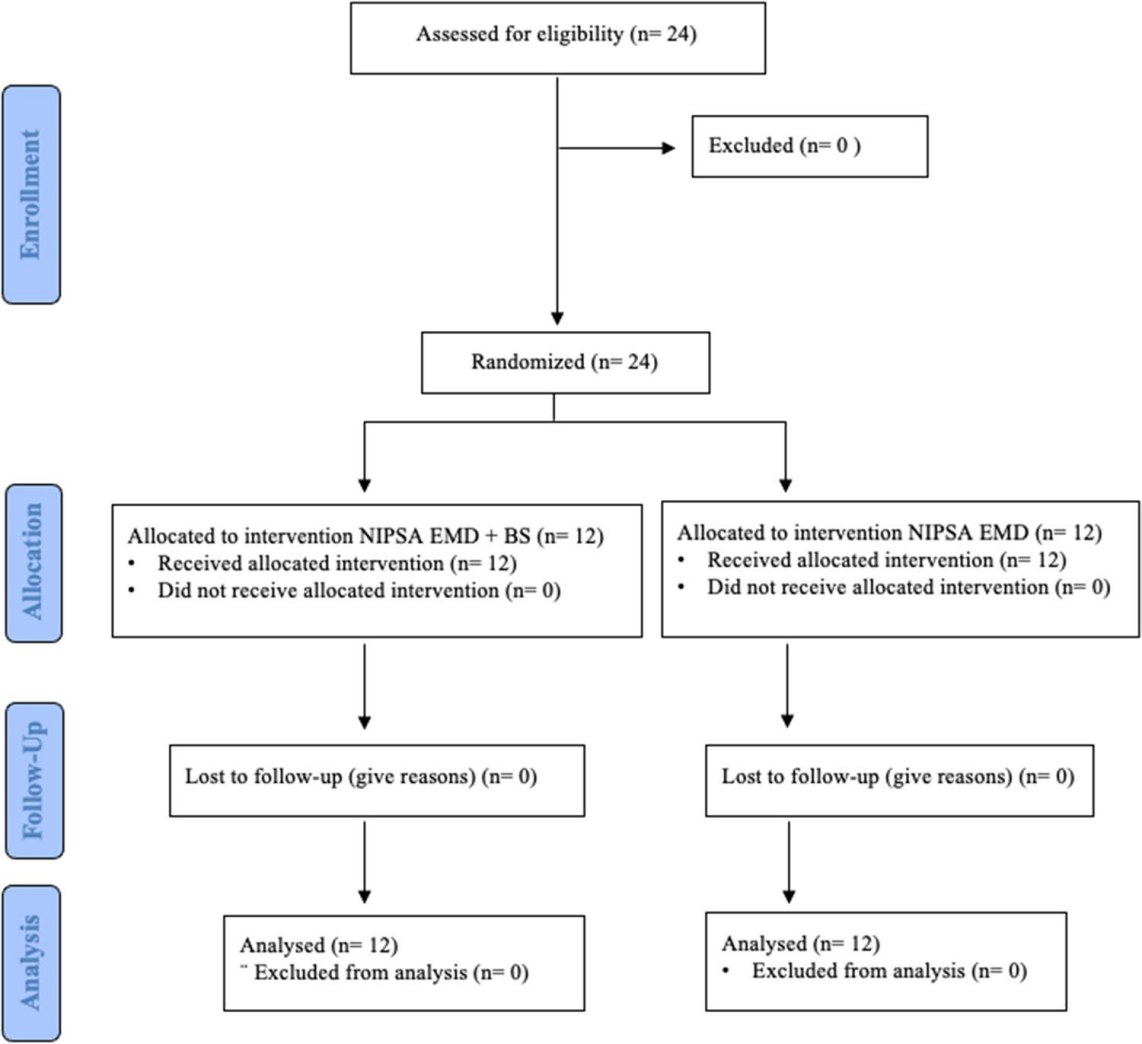
Flow chart of the study

Patients who met the inclusion criteria were randomly assigned to one of the experimental groups (http://www.graphpad.com/quickcalcs/randomize1.cfm). All periodontal defects were surgically assessed using NIPSA and affected root surfaces treated with enamel matrix protein derivates (EMD; Institut Straumann, AG, Basel, Switzerland). Periodontal defects were filled with a combination of EMD and BS in the control group (NIPSA EMD + BS), while the test group (NIPSA EMD) did not receive BS. All interventions were made by the same surgeon (JAMR). Treatment allocation was disclosed to the surgeon after surgical debridement of the defect and root surface treatment. An experienced researcher (AJOR) made all measurements. He was blinded to the technique used and attended a preliminary calibration session in 20 patients not involved in the experimental procedures, reporting a single-score intraclass correlation of 0.902 (95% CI; 0.773–0.96) for CAL and 0.918 (95% CI; 0.809–0.967) for PD. All patients, also blinded, were informed about the procedures and gave written informed consent. The study flow chart is shown in Fig. 1.

### Participants and defect eligibility

The inclusion criteria were (1) diagnosis of periodontal disease (periodontitis stage III and IV, grade A [12]; (2) non-smokers and former smokers who quit smoking at least 1 year previously [13]; (3) previous treatment for periodontal disease using non-surgical periodontal treatment (completion of step I and II of periodontal therapy; Supplementary table 1) [14, 15]; (4) compliance with periodontal maintenance and oral hygiene (FMPS at baseline ≤ 30%) [16–18]; (5) active residual pockets (positive bleeding on probing; BoP) associated with intrabony defects that were not resolved with non-surgical treatment after 4–6 months of maintenance [14, 15]; (6) periodontal lesions with probing depth (PD) > 6 mm and extension of the intrabony defect > 3 mm (pre-diagnosis clinically (periodontal probe) and radiographically, and corroborated intra-surgically); (7) isolated, deep combined intra-suprabony periodontal defects; (8) 1 and/or 2 walls intrabony defects, at least, in some aspect of its three-dimensional configuration, always involving the buccal aspect (pure 3-wall defects, pre-diagnosed clinically and radiographically, and corroborated intra-surgically were excluded); and (9) defects with a supra-alveolar component (interproximal bone crest distance to the cement-enamel junction limit (BC-CEJ) ≥ 4 mm).

All inclusion criteria involving the configuration of periodontal defects were evaluated in the pre-surgical phase, and re-evaluated intra-surgically. Exclusion criteria were (1) systemic disease contraindicating periodontal surgery, (2) smokers, and (3) third molars or teeth with incorrect endodontic or restorative treatment.

### Interventions

#### Pre-surgical procedures

One to two weeks pre-surgery, periodontal tissues were conditioned for surgical treatment. The residual periodontal pocket was treated using ultrasonic scaler tips and microcurettes, instrumenting only the exposed root surface and the first 3 mm of the root beneath the periodontal pocket [9–11]. Teeth with grade > 1 mobility were splinted. Surgery was not begun until there was minimal or no marginal inflammation and the marginal tissue had a fibrous tone to ensure correct manipulation.

#### Surgical procedures

All interventions were made using 4 to 10 × magnification (Labomed Microscope. LABO AMERICA INC.). Tissue was handled using 4 × magnification and root surface detoxification using 10 × magnification to ensure correct elimination of any reservoir of bacterial plaque. The surgical area was anesthetized with articaine/epinephrine 1:100,000 (Ultracain, Laboratorios Normon, S.A. Madrid, Spain).

The periodontal defect was accessed using NIPSA [9–11]. A single horizontal or oblique incision was made in the mucosa located on the cortical bone tissue, apically at the edge of the bone crest that delimited the intrabony defect and as far as possible from the marginal tissues. The mesiodistal extension of the incision, although reduced, was sufficient to allow access to the defect, expose its limits, and allow correct debridement of the periodontal pocket and the application of biomaterials. Tissue coronal to the incision was raised to full thickness from the incision line to expose the bone peaks delimiting the intrabony defect. The interproximal soft tissue was pulled coronally to expose the supra-alveolar component of the defect, always maintaining its structural integrity. If the defect had a lingual component, the area was accessed using buccal access.

**Fig. 2:**
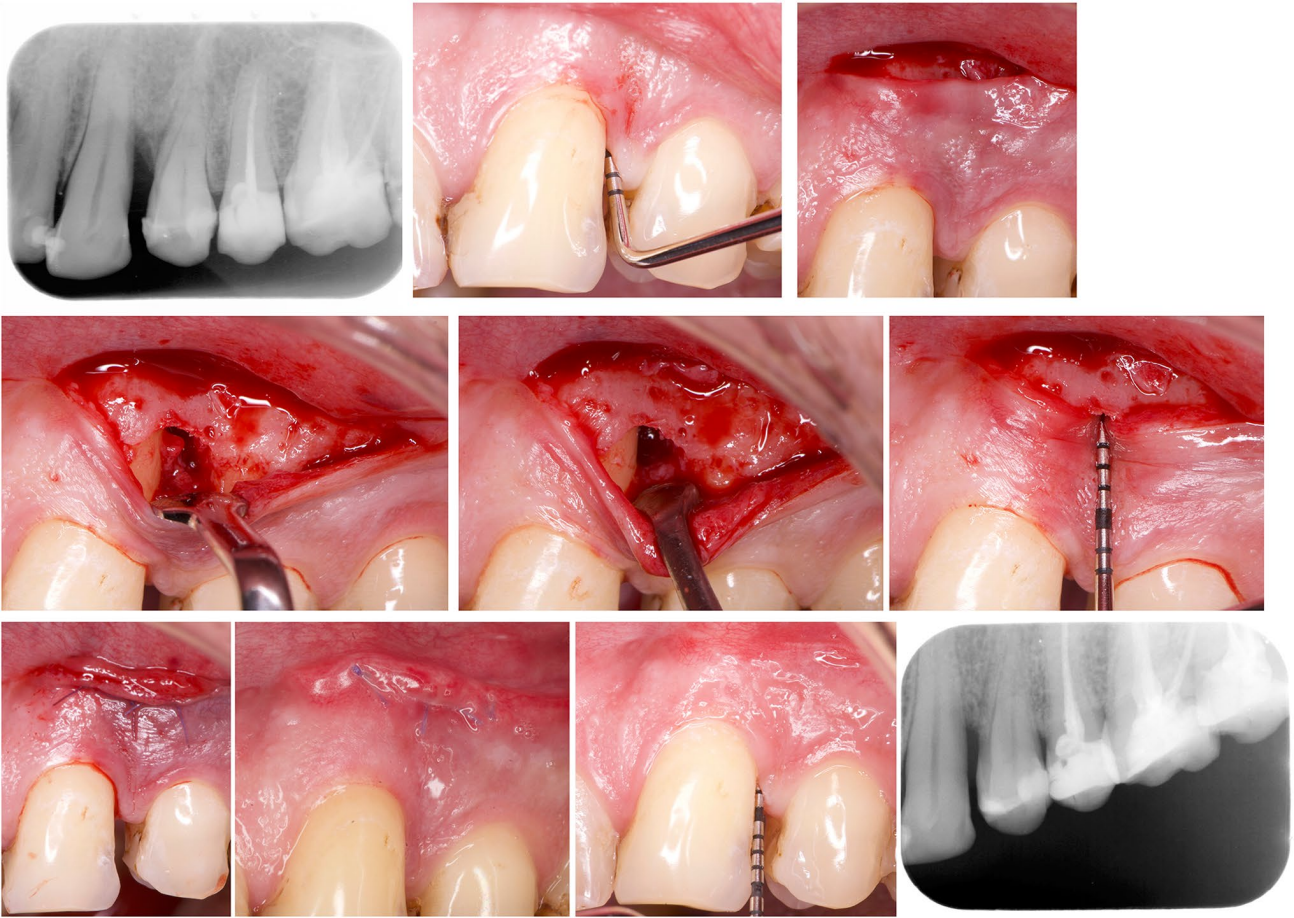
NIPSA EMD without BS. **a** Periapical diagnostic X-ray. **b** Interproximal PD before surgery. **c** Apical incision. **d** Elevation of tissue coronal to the incision to expose the bone peaks delimiting the defect and coronal traction of the interproximal tissue to expose the supra-alveolar component. Internal appearance of the periodontal pocket around the affected root surface. **e** Intrabony component of the defect after debridement of granulation tissue and removal of the periodontal pocket. Intrabony defect configuration: 3-wall component in deepest aspect and 1-wall in coronal aspect (non-contained defect). **f** Probe of the 3-wall component of the intrabony defect. **g** Suture and preservation of marginal tissue. **h** Primary wound closure at 1 week post-surgery. **i**–**j** One-year follow-up. Improvement in residual PD and interproximal soft tissue. Periapical X-ray shows complete bone filling (no standardized radiographs)

**Fig. 3:**
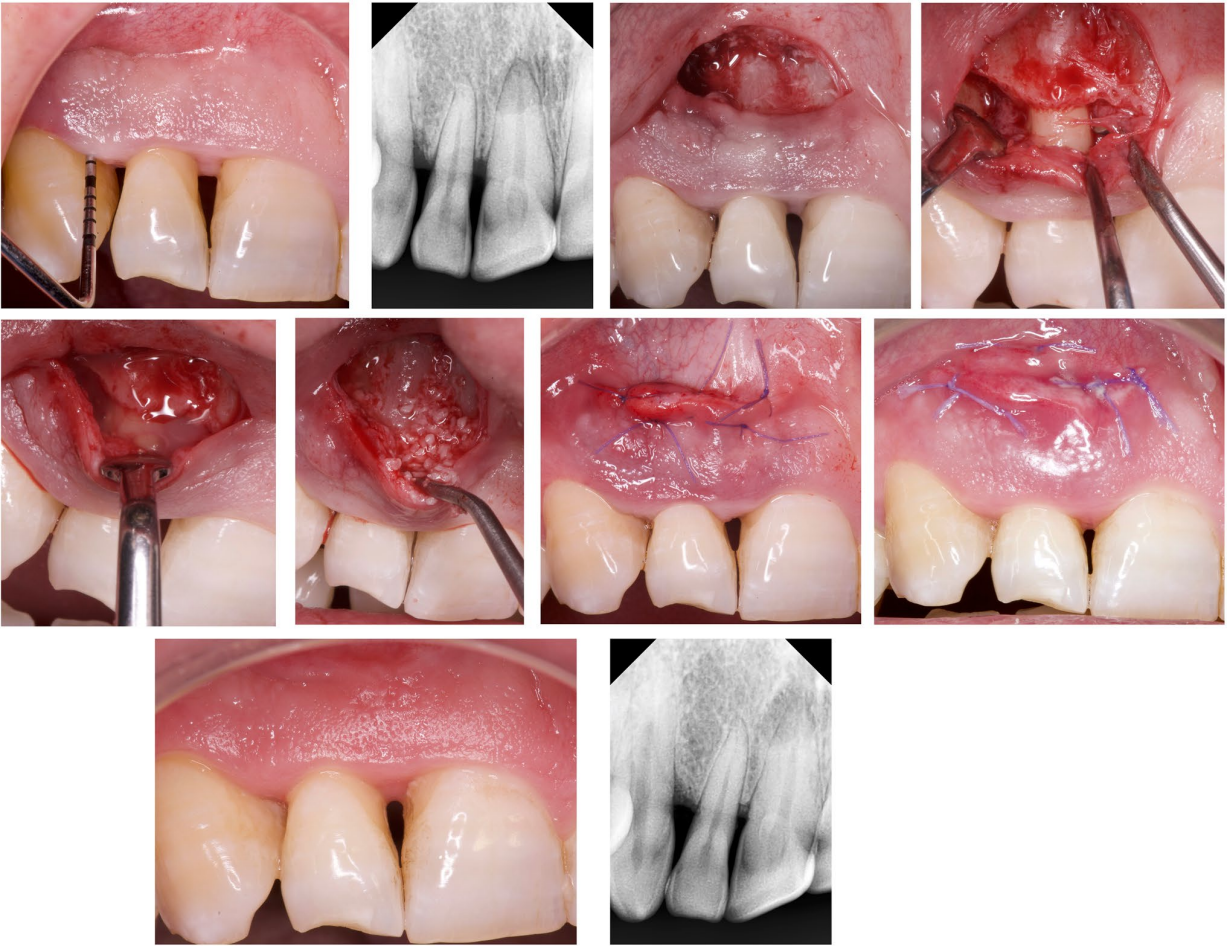
NIPSA + EMD + BS. **a**–**b** Interproximal PD and periapical X-ray before surgery. Deep combined non-contained intrabony and supra-alveolar periodontal defect. Soft tissue superficial fibrous tone after the pre-surgical procedures, and “red-wine” translucent from the deep aspect. **c** Apical incision in the mucosa located on the cortical bone tissue, as far as possible from the marginal tissues. **d** Elevation of the tissue coronal to the incision to expose the bone peaks delimiting the non-contained intrabony defect and coronal traction of the interproximal tissue to expose the supra-alveolar component. **e**–**f** Biomaterials application. **g** Suture. **h** Primary wound closure and soft tissue preservation 1 week post-surgery. **i**–**j** One-year follow-up. Soft tissue preservation and healthy aspect. Periapical X-ray (no standardized radiographs)

During defect debridement, coronal tissue was protected with a micro-periostotome and frequently irrigated with saline solution. The granulation tissue was disinserted from the bone walls of the defect using micro-minicurettes and, together with the periodontal pocket, was sectioned from the base of the marginal and interproximal soft tissue by surgical microblades or microscissors, and removed. Dental plaque and calculus deposits were removed from the root surface with ultrasonic scaler tips and micro-minicurettes. 24% Ethylenediaminetetraacetic acid (EDTA) (PrefGel. Straumann, Basel, Switzerland) was applied to the root surface and, after 2 min, irrigated with abundant saline solution, and EMD was applied to the root surface. BS was not applied in the test group (Fig. 2). In the control group (Fig. 3), the defect was filled with a mixture of EMD + BS (xenograft bone substitute, Cerabone, Botiss Biomaterials GmbH, Berlin, Germany). The incision was sutured with a double suture line, the first with horizontal internal mattress sutures to approximate the connective tissue of the mucous edges of the incision, and a second line of simple sutures (PGA 6/0. Hu-Friedy, Frankfurt, Germany).

#### Post-surgical procedures

All patients received amoxicillin 500 mg every 8 h for 5 days (azithromycin 500 mg once daily for 3 days in patients allergic to amoxicillin). Postoperative pain and inflammation were controlled using ibuprofen 600 mg (or acetaminophen 1 g in patients allergic to ibuprofen). Patients rinsed with 0.2% chlorhexidine twice daily for 1 week, without mechanical hygiene on the surgical area. Sutures were removed at 1 week, and patients were instructed to mechanically clean the area using a soft brush and apicocoronal brushing. Maintenance visits were made at 1, 2, 3, and 4 weeks, and 3, 6, and 12 months, including supportive periodontal therapy and oral hygiene reinforcement.

### Primary and secondary outcome measures

#### Primary outcome: interproximal clinical attachment level gain

Secondary outcomes: residual PD, PD reduction, recession (REC), location of the tip of the papilla (TP), width of the keratinized tissue (KT), wound closure (WC), supra-alveolar attachment gain (SUPRA-AG).

#### Clinical measurements

Measurements were made immediately before surgery and at 1 year using a millimeter periodontal probe (PCP UNC 15, Hu-Friedy, Frankfurt, Germany) taking the largest measurement and the nearest millimeter:

(1) BoP; (2) CAL, measured in the interproximal space from the CEJ to the bottom of the pocket (BP); (3) Interproximal PD, measured in the interproximal space from the gingival margin to the BP; (4) REC, measured on the buccal face, at the level of the central axis of the tooth, from the CEJ to the gingival margin; (5) TP, taking as a reference the central axis of the tooth, the distance from the CEJ in the zenith of the tooth to the vertex of the papilla, which is positive when the papilla is located coronally to the CEJ; (6) KT, measured from the gingival margin to the mucogingival line through the central axis of the tooth.

#### Intrasurgical measures

To define the morphology of the defect, the following parameters were recorded immediately after debridement of the periodontal lesion: (1) intrabony component of the defect: distance from the coronal limit of the interproximal bone crest (BC) to the bottom of the bone defect (BD), (2) 3-wall component of the intrabony defect: distance from the coronal limit of the 3-wall defect to the BD; (3) configuration of the intrabony defect according to the number of walls (3 walls; 2 walls; 1 wall), (4) interproximal BC-CEJ, and (5) interproximal supra-alveolar soft tissue (SUPRA-ST): distance from BC to TP.

#### Post-surgical measures

Three types of WC were recorded at one week [10]: WC = 2: first intention healing, or complete closure of the incision line, with or without a minimum fibrin line at the incision level; WC = 1. Second intention healing, or incomplete closure, with fibrin clotting in the incision area; WC = 0. Incomplete closure, with tissue necrosis in the interproximal area.

One year after surgery, the SUPRA-AG [11] was calculated. SUPRA-AG is the result of subtracting the value of CAL in the follow-up from the BC-CEJ distance, measured intra-surgically in the interproximal aspect. A positive value indicated that the attachment gained was above the intrabony defect, while a negative value indicated that the intrabony defect was not completely resolved.

### Statistical analysis

Patients contributed one defect site. Therefore, the patient was considered as the statistical unit. The sample size (*n* = 12 per group) was calculated for two paired means, repeated in two groups, using CAL gain values, and accepting an alpha risk of 0.05, a beta risk of 0.20 (power 0.8) in a two-sided test, to recognize a minimum difference of ≥ 1.25 units as statistically significant. A common standard deviation of 1.45 and a correlation coefficient between the baseline and final measurements of 0.75 were assumed. A dropout rate of 10% was anticipated. CAL gain values were obtained from a pilot study carried out in five patients per group.

In the descriptive analysis, values were expressed as mean ± SD. The Shapiro–Wilk normality test and the Levene test for equality of variances were used for quantitative variables.

Between-group comparisons were made using the Student’s *t*-test when there was normality and equality of variances and the Mann–Whitney test when there was not.

Values at baseline and at 1 year were compared using the paired *t* test for normally distributed values with equal variances and the Wilcoxon test for non-normally distributed values and/or those with unequal variances.

Qualitative variables were compared using contingency tables and Fisher’s exact test. A value of *p* < 0.05 was considered statistically significant. The statistical analysis was performed using the R statistical package.

## Results

### Experimental population and defect characteristics

Twenty-four patients were invited to participate in the trial: 12 were treated with NIPSA EMD without BS (mean age 46.50 ± 10.47, 6 female) and 12 with NIPSA EMD + BS (mean age 50.33 ± 9.02, 3 female). All patients completed the study protocol and attended review and maintenance appointments.

The characteristics of the study patients and the defects treated are shown in Table 1. No significant differences between-group differences were found: the defects were most often located in the upper arch (ratio 2:1), and 83% were in monoradicular teeth.

**Table 1:** Patient and defect characteristics

	NIPSA EMD (*n* = 12)	NIPSA EMD + BS (*n* = 12)	*P* value
Study population			
Sex (male/female)	6/6	9/3	0.40 a
Age (Years) (mean ± SD)	46.50 ± 10.47	50.33 ± 9.02	0.347 c
Dental arch (upper/lower)	8/4	8/4	1.00 a
Tooth type (Incisors/canines/premolars/molars)	9/1/1/1	5/5/0/2	0.199 a
Defect morphology measurements (mm)			
CEJ-BC (mean ± SD)	4.83 ± 1.19	5.33 ± 2.06	0.802 b
INTRA	5.92 ± 1.83	6.08 ± 1.88	0.813 b
SUPRA-ST	6.67 ± 1.37	6.42 ± 1.62	0.553 b
Intrabony defect configuration			
1/3-wall	8	8	
2/3-wall	-	2	
1/2-wall	2	-	
1/2/3-wall	1	-	
1-wall	1	2	

*NIPSA* non-incised papillae surgical approach, *EMD* enamel matrix protein derivates, *BS* bone substitutes, *CEJ* cement-enamel junction, *INTRA* intrabony defect, *SUPRA-ST* interproximal supra-alveolar soft tissue

^a^Fisher exact test

^b^Mann–Whitney test

^c^*t*-test

All residual periodontal pockets treated were associated with deep combined intrabony (NIPSA EMD group 5.92 ± 1.83 mm; NIPSA EMD + BS 6.08 ± 1.88 mm) and supra-alveolar defects (BC-CEJ: NIPSA EMD group 4.83 ± 1.19 mm; NIPSA EMD + BS group 5.33 ± 2.06 mm). In all defects, the intrabony component had a non-contained configuration, and 91.66% presented 1 wall somewhere in the three-dimensional configuration.

### Clinical outcomes

Periodontal parameters at baseline and 1 year are shown in Tables 2 and 3.

**Table 2:** Clinical parameters (mm)

	Baseline	1 year	1-year change	*P* value
PD		Residual PD	PD reduction**	
NIPSA EMD	10.75 ± 2.77	2.50 ± 0.67	8.25 ± 2.70	< 0.001^b^
NIPSA EMD + BS	9.50 ± 2.43	2.67 ± 0.78	6.83 ± 0.81	< 0.001^b^
P VALUE	0.168^a^		0.090^a^	
CAL			**CAL gain^**	
NIPSA EMD	11.33 ± 2.87	3.00 ± 0.95	8.33 ± 2.74	< 0.001^b^
NIPSA EMD + BS	10.42 ± 3.40	3.33 ± 1.23	7.08 ± 2.68	< 0.001^c^
* P* value	0.306^a^		0.17^a^	
REC*				
NIPSA EMD	0.58 ± 0.67	0.83 ± 0.72	− 0.25 ± 0.45	0.250^b^
NIPSA EMD + BS	0.83 ± 1.19	1.00 ± 1.13	− 0.17 ± 0.58	0.375^b^
* P* value	0.849^a^		0.771^a^	
TP#				
NIPSA EMD	2.50 ± 1.62	2.83 ± 1.64	− 0.33 ± 0.49	0.125^b^
NIPSA EMD + BS	2.17 ± 1.27	2.67 ± 1.23	− 0.45 ± 0.52	0.031^b^
* P* value	0.581^d^		0.585^a^	
KT				
NIPSA EMD	4.25 ± 1.60	4.25 ± 1.48	0.00 ± 0.43	1.00^b^
NIPSA EMD + BS	4.08 ± 1.56	4.17 ± 1.47	− 0.08 ± 0.67	0.813^b^
* P* value	0.799^d^		0.718^a^	
WC (1 week)	2	< 2		
NIPSA EMD	11	1		
NIPSA EMD + BS	10	2		
* P* value	1.0^e^	1.0^e^		
SUPRA-AG¨				
NIPSA EMD			1.83 ± 1.11	
NIPSA EMD + BS			2.00 ± 1.76	
* P* value			0.784^a^	

*NIPSA* non-incised papillae surgical approach, *EMD* enamel matrix protein derivates, *BS* bone substitutes, *PD* probing depth, *CAL* clinical attachment level, *REC* recession, *TP* location of the tip of the papilla, *KT* keratinized tissue, *SUPRA-AG* supra-alveolar attachment gain, *mm* millimeters, *NS* not significant, *p* > .05

^*^Negative value in REC Change indicates increased recession. #Negative value in TP change indicates papilla coronal displacement. ^CAL change = CALgain. ¨Positive value in SUPRA-AG indicates complete resolution of the intrabony defect. **PD change = PD reduction

^a^Mann–Whitney test

^b^Wilcoxon test

^c^Paired *t*-test

^d^*T*-test

^e^Fisher exact test

**Table 3:** Frequency distribution of probing depth reduction, clinical attachment gain, and residual probing depth in study groups

	NIPSA EMD			NIPSA EMD + BS		
mm	PD reduction	CAL gain	Residual PD	PD reduction	CAL gain	residual PD
2	-	-	7 (58.33%)	-	-	6 (50.0%)
3	-	-	4 (33.33%)	-	-	4 (33.33%)
4	-	-	1 (8.33%)	1 (8.33%)	1 (8.33%)	2 (16.66%)
5	1 (8.33%)	1 (8.33%)	-	5 (41.66%)	3 (25.0%)	-
6	2 (16.66%)	2 (16.66%)	-	2 (16.66%)	3 (25.0%)	-
7	4 (33.33%)	4 (33.33%)	-	-	1 (8.33%)	-
8	1 (8.33%)	1 (8.33%)	-	1 (8.33%)	1 (8.33%)	-
9	1 (8.33%)	-	-	1 (8.33%)	1 (8.33%)	-
10	-	1 (8.33%)	-	-	-	-
11	1 (8.33%)	1 (8.33%)	-	-	-	-
12	-	-	-	2 (16.66%)	2 (16.66%)	-
13	2 (16.66%)	2 (16.66%)	-	-		-
TOTAL	12	12	12	12	12	12

*NIPSA* non-incised papillae surgical approach, *EMD* enamel matrix protein derivates, *BS* bone substitutes, *PD reduction* probing depth reduction, *CAL gain* clinical attachment level gain, *residual PD* residual probing depth

At 1 week, 87.5% of cases had complete closure of the incision line (WC = 2), with no cases of necrosis of the interproximal tissue (WC = 0). One case in the NIPSA EMD group and two in the NIPSA + EMD + BS group showed fibrin on the incision line (WC = 1).

All sites showed positive BoP before surgery and negative BoP at 1 year. Both groups showed significant PD reduction (*p* < 0.001) at 1 year, with no significant between group differences. The PD reduction was > 6 mm in 75% of cases in the NIPSA + EMD group, and 33.33% of cases in the NIPSA EMD + BS group. The residual PD was < 5 mm in both groups (NIPSA EMD 2.50 ± 0.67 mm; NIPSA EMD + BS 2.67 ± 0.78 mm). The residual PD was 2 mm in 58.33% of cases receiving NIPSA EMD and 50% of cases receiving NIPSA EMD + BS. The residual PD was 4 mm in one case in the NIPSA EMD group, and two cases in the NIPSA EMD + BS group. Similarly, CAL gain was significant in both groups (NIPSA EMD group 8.33 ± 2.74 mm; NIPSA EMD + BS group 7.08 ± 2.68; *p* < 0.001), with no significant between-group differences. CAL gain was > 6 mm in 75% of cases treated with NIPSA + EMD and 41.66% of cases treated with NIPSA EMD + BS.

No significant changes were found in the REC and KT in either group. TP showed significant changes in the NIPSA EMD + BS group (*p* = 0.031) at 1 year, with a coronal displacement of the papilla of 0.45 ± 0.52 mm, but there were no significant between-group differences in the mean gain.

In both groups, there was CAL gain in the supra-alveolar component (SUPRA-AG: NIPSA EMD 1.83 ± 1.11 mm; NIPSA EMD + BS 2.00 ± 1.76 mm), with no significant between-group differences. Therefore, the intrabony component of the defect was completely resolved in all cases.

## Discussion

We used NIPSA and EMD, with and without BS, in the treatment of deep periodontal lesions. The aim was to evaluate the influence of BS in clinical response of periodontal tissues, which may provide three-dimensional stability to soft tissues in complex defects with no bone peaks or bony wall support that may prevent gingival tissue from collapsing.

Therefore, defect eligibility centered on deep, non-contained intrabony defects with 1 and/or 2 walls and supra-alveolar type defects. Studies show that supra-alveolar interproximal defects account for > 91% of periodontal defects and intrabony defects for < 9% [19–22] and that 84% of intrabony defects have greater involvement of the buccal bone peak than the lingual [23]. Likewise, about 35% of intrabony defects are 1 wall and 50% 2 walls [23]. Although supra-alveolar defects are the most common, few studies have evaluated the results of periodontal reconstructive surgery in treating these defects [11, 18, 24–27], with most studies focusing on intrabony defects [28, 29].

A baseline FMPS ≤ 25% was established in the inclusion criteria of other periodontal regenerative surgery studies [30–32]. In periodontitis stage III and IV patients, it is common to observe black triangles, dental crowding and other local factors that complicate complete control of bacterial plaque. Therefore, we used a less rigorous FMPS (≤ 30%) as did similar studies [17, 18].

Based on scientific evidence, surgery is indicated in residual deep pockets with positive BoP that are not resolved after periodontitis step I–II therapy [14, 15]. However, BoP should be controlled before periodontal regenerative surgery [33]. We included defects with a complex configuration to evaluate and achieve the study objectives. The complexity of the defects and the depth of the residual pockets mean that complete control of inflammation of the pocket is difficult. In the pre-surgical phase, the marginal tissue was pre-conditioned in a minimally invasive manner in order to achieve a fibrous tone and improve surgical handling of the tissue. However, the deep part of the periodontal pocket, where previous non-surgical treatment was not efficacious, was not treated, in order to avoid repeated and unnecessary trauma to the wall of the periodontal pocket (supra-alveolar defects) and prevent secondary recession of the soft tissue. This apical area was later treated during periodontal surgery [9–11]. Therefore, the persistence of inflammation in deep areas (positive BOP) at baseline is common. As in various studies, regenerative periodontal surgery was carried out in residual pockets with a positive BoP [18, 26, 34, 35].

Studies suggest periodontal regeneration requires several conditions: primary intention healing, space provision for clot stabilization, and wound stability during healing [36–38]. Periodontal surgery is challenging due to the differing tissues involved: the mineralized root surface and the gingival tissue [39], which may hinder adequate healing because primary intention closure is not possible [37]; mechanical forces constantly act on marginal tissues during healing [39]; there is a tendency for soft tissues repositioned on a hard, smooth convex root surface to collapse [26, 39, 40]; and wound healing is exposed to the oral cavity, where external agents, such as dental plaque [41] and smoking products [42], may have a negative influence.

NIPSA was developed to promote optimal periodontal healing. Using a single mucosal incision, apical to the periodontal defect, the aim is complete wound closure of the incision line and primary intention healing. The coronal tissue is raised from the incision line until the bone peaks are displayed and the SUPRA-ST is pulled coronally to expose the supra-alveolar component of the defect without a marginal incision, which maintains their structural integrity and the architecture of the interproximal soft tissues, preventing them from collapsing over the underlying bone peaks. This helps maintain space for periodontal regeneration and stability during wound healing and the establishment of early brushing techniques by the patient for more effective removal of dental plaque, the etiological factor for periodontal disease.

EMD, whose regenerative properties are widely demonstrated [43], was used to promote regeneration in the complex defects included. These types of defects are a challenge in periodontal regenerative surgery due to the difficulty of periodontal ligament, cement, and alveolar bone progenitor cells in recolonizing the supra-alveolar and non-contained areas that are far from the area to be regenerated [37]. However, EMD lacks the mechanical properties to support soft tissues [44], and therefore, its use is associated with that of BS, which enhances the clinical outcome of EMD [45–47], improving its space-making potential, by preventing the collapse of the overlying soft tissues into the regeneration area [48, 49]. However, studies show that slowly resorbing BS may interfere with wound healing, bone formation, and periodontal regeneration [50–53]. Various RCTs found no beneficial effects on clinical outcomes (CAL gain, PD reduction, and REC change) of the addition of EMD to BS in the treatment of intrabony defects with flap designs with intrasulcular incisions [54]. Furthermore, the use of EMD alone in the treatment of supra-alveolar defects improved the healing of soft tissues [55]. Others studies [56, 57] suggested that the use of EMD alone in non-contained intrabony defects is not recommended. However, all of these studies performed the approach to periodontal defects with intrasulcular incisions and marginal access. In this study, we performed the approach to the periodontal defect through an apical access and we evaluated the capacity of this new approach in maintaining the stability and structural integrity of the marginal soft tissues.

In both groups, PD reduction and CAL gain were significant (< 0.001) without between-group differences. The residual PD was < 5 mm in all defects, although residual PD = 4 was recorded in twice as many cases (16.66%) when BS was used. The CAL gain was > 6 mm in 75% of cases treated without BS versus 41.66% in cases with BS.

Evidence suggests that residual PD > 4 mm is a risk factor for periodontal disease progression [58]. However, in the surgical treatment of periodontal defects, it is important not only to resolve the periodontal pocket, but also to achieve a CAL gain coupled with minimal soft tissue recession [6, 59]. Tissue collapse reduces, and even eliminates, the periodontal pocket but limits the CAL gain to the intrabony component [26, 27]. Therefore, PD reduction or CAL gain should not be evaluated as a separate parameter [59]. To evaluate the success of surgery, PD reduction may be associated with CAL gain added to soft tissue preservation (REC and TP change) or improvement. The preservation of the SUPRA-ST allows the space and stability for the clot to be maintained and the resolution of the supra-alveolar component of the periodontal pocket promoted at the expense of reattachment and not the recession of tissues [11]. This results in trying to “recover” periodontal clinical attachment through tissue preservation using surgery.

Our clinical results show, together with the good values of PD reduction and CAL gain, the preservation of soft tissues (REC, TP, KT) in both groups without significant between-group differences. In addition, when BS was used, there was an improvement in interproximal soft tissues, with a significant (*p* < 0.005) papilla coronal displacement (TP change), possibly due to the intrasurgical coronal traction of the interproximal soft tissue above its baseline situation, and then maintained with the help of BS.

Periodontal prognoses depend on the interproximal attachment, and therefore, interproximal CAL gain may improve the periodontal disease status [6]. CAL gain is recorded taking CEJ as a reference. SUPRA-AG, a recently introduced parameter, helps situate the CAL gain with respect to the baseline interproximal bone peak and provides information that may be used to assess periodontal surgery and the prognosis of the treated lesion: It indicates (1) the resolution of the intrabony defect or the presence of a residual intrabony defect, (2) the CAL gain into the supra-alveolar component (positive SUPRA-AG), and (3) helps determine if the residual PD is intrabony (negative SUPRA-AG). No defect treated in this study had a negative SUPRA-AG. In the same way as a residual PD ≥ 5 mm [60], the persistence of an intrabony defect is associated with a high risk of progression of periodontal disease [61]. SUPRA-AG may be achieved only if the soft tissue is preserved. Although TP change was only significant in the NIPSA EMD + BS group, the result in the NIPSA EMD group was similar (− 0.45 ± 0.52 mm vs − 0.33 ± 0.49 mm; *p* = 0.58), and due to the preservation of interproximal soft tissue (TP), a positive SUPRA-AG was achieved (2.00 ± 1.76 mm; 1.83 ± 1.11 mm; *p* = 0.784). These outcomes were similar to those found in a study of NIPSA and the use of BS + EMD in the treatment of periodontal defects with a supra-alveolar component (TP change − 0.4 ± 0.5 mm; SUPRA-AG 1.9 ± 1.74 mm) (Moreno et al. 2019).

Soft tissue management with NIPSA (apical incision and access) differs from other regenerative periodontal techniques (intrasulcular incisions and marginal access). The resulting residual PD with NIPSA (2.50 ± 0.67 mm and 2.67 ± 0.78 mm for test and control procedures) is similar to that in other studies of regenerative periodontal surgery (2.48 ± 0.65 mm [25], 2.75 ± 0.75 mm [62], 2.83 ± 0.74 mm [63], 2.9 ± 0.8 mm [30], 2.5 ± 0.6 mm [31]). However, CAL gain with NIPSA seems to be superior (8.33 ± 2.74 mm and 7.08 ± 2.68 mm for test and control procedures) than with intrasulcular incision procedures in which the best CAL gain was 6.83 ± 2.51 mm [62] and 6.5 ± 2.4 mm [32]. The explanation may be in the iGR results. Intrasulcular incision results in a significant iGR (0.9 ± 1.1 mm [64], 1.1 ± 1.1 and 0.8 ± 1.2 mm [65], 1.1 ± 1.1 and 1.0 ± 1.1 [26]). NIPSA avoids marginal tissue incisions with the objective of maintaining wound stability and counteracting post-surgical soft tissue collapse (interdental soft tissue coronal displacement of 0.33 ± 0.49 for test group, *p* > 0.05). Furthermore, using BS, interdental soft tissues may be stabilized at a more coronal location and BS seems to result in significant interdental soft tissue improvement (0.45 ± 0.52 for control group, *p* < 0.05). The results of this study showed that interdental soft tissue preservation, or even improvement, results in an increased CAL gain.

NIPSA is indicated in the treatment of isolated, deep residual periodontal pockets associated with non-contained bone defects, and intrabony defects lacking the buccal wall (1 or 2 walls) and/or with a supra-alveolar component [11]. NIPSA is especially indicated in pockets located in the anterior sector, where esthetic concerns require optimal soft tissue preservation and accessibility from the buccal aspect is good. However, if the defect has an extensive lingual component, this may affect the results due to the reduced visibility for the treatment of the lingual root surface [10].

## Conclusions

NIPSA provides ideal conditions to obtain periodontal pocket resolution and clinical attachment gain associated with preservation of the soft tissue and is a valid surgical approach in the treatment of deep, isolated non-contained periodontal defects with a supra-alveolar component, where supra-soft tissue stability may be compromised. Preservation and, even improvement, of the interproximal soft tissue permits supra-alveolar attachment gain. Although a significant papilla improvement was only recorded when BS was used, there were no significant clinical differences in the non-BS group. Long-term results for this technique are needed to confirm the present findings.

## Supplementary Information

Below is the link to the electronic supplementary material.

Supplementary file1 (JPG 350 KB)
