# Supplementary figures and images for: Apical approach in periodontal reconstructive surgery with enamel matrix derivate and enamel matrix derivate plus bone substitutes: a randomized, controlled clinical trial
Source: Clin Oral Investig. 2021 Nov 17;26(3):2793–805. doi: 10.1007/s00784-021-04256-1 (PMC8898230; doi:10.1007/s00784-021-04256-1)

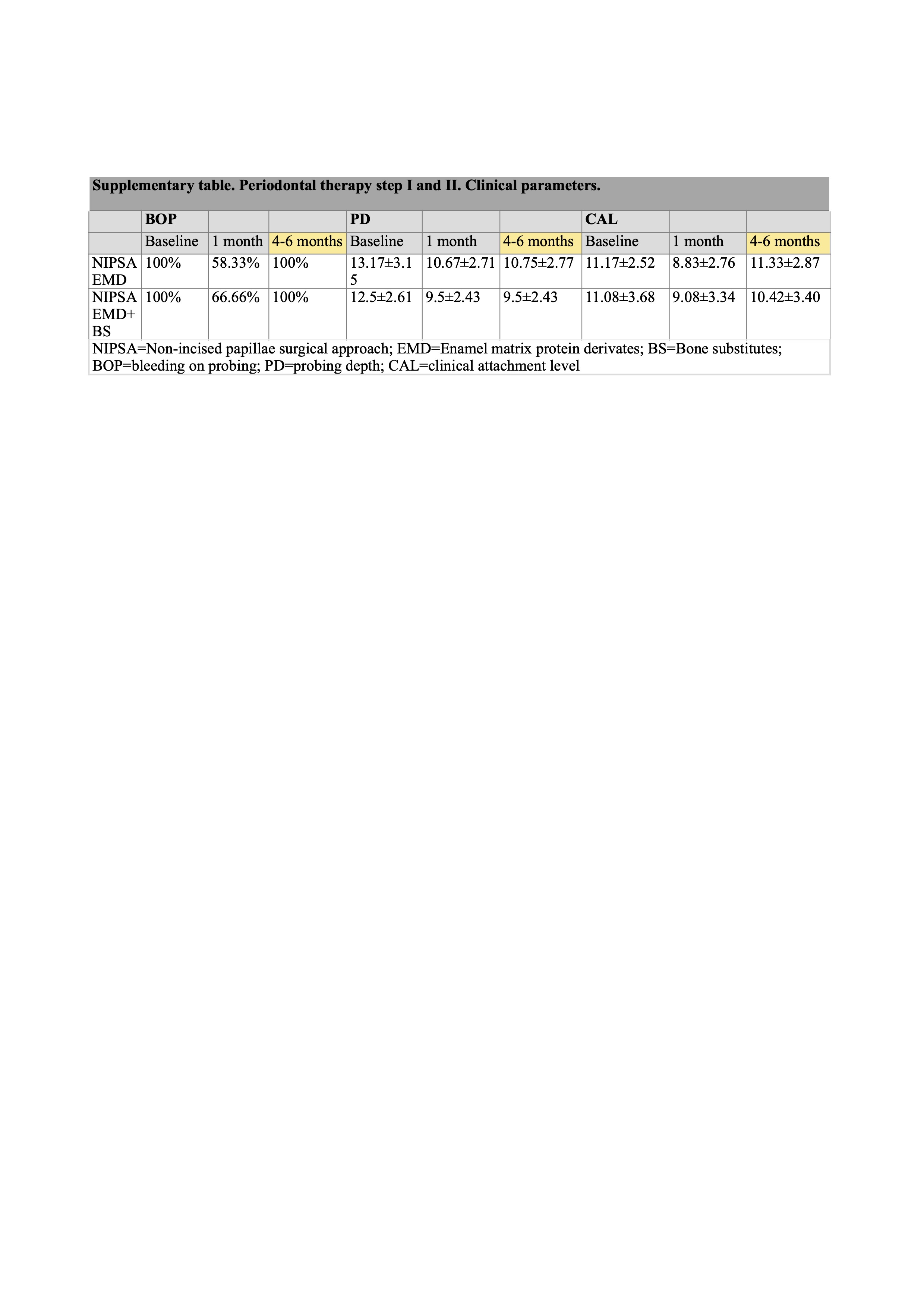

Supplement: Supplementary file 1 — Supplementary file1 (JPG 350 KB) [file 784_2021_4256_MOESM1_ESM.jpg]
